# The role of mutations associated with familial neurodegenerative disorders on blood–brain barrier function in an iPSC model

**DOI:** 10.1186/s12987-019-0139-4

**Published:** 2019-07-15

**Authors:** Moriah E. Katt, Lakyn N. Mayo, Shannon E. Ellis, Vasiliki Mahairaki, Jeffrey D. Rothstein, Linzhao Cheng, Peter C. Searson

**Affiliations:** 1 Institute for Nanobiotechnology, Johns Hopkins University, 100 Croft Hall, 3400 North Charles Street, Baltimore, MD 21218 USA; 2 Department of Materials Science and Engineering, Johns Hopkins University, Baltimore, MD USA; 3 Department of Biostatistics, Johns Hopkins University School of Public Health, Baltimore, MD USA; 4 Department of Neurology, Johns Hopkins University School of Medicine, Baltimore, MD USA; 5 Institute for Cell Engineering, Johns Hopkins University School of Medicine, Baltimore, MD USA; 6 Department of Neuroscience, Johns Hopkins University School of Medicine, Baltimore, MD USA; 7 Department of Biomedical Engineering, Johns Hopkins University School of Medicine, Baltimore, MD USA

**Keywords:** Induced pluripotent stem cells, Human brain microvascular endothelial cells, Blood–brain barrier, Differentiation, Neurodegenerative disease

## Abstract

**Background:**

Blood–brain barrier dysfunction is associated with many late-stage neurodegenerative diseases. An emerging question is whether the mutations associated with neurodegenerative diseases can independently lead to blood–brain barrier (BBB) dysfunction. Studies from patient-derived induced pluripotent stem cells suggest that mutations associated with neurodegenerative disease are non-cell autonomous, resulting in gain of toxic function in derived neurons and astrocytes. Here we assess whether selected mutations associated with neurodegenerative diseases can contribute to impairment of the blood–brain barrier.

**Methods:**

We assessed barrier function of confluent monolayers of human brain microvascular endothelial cells (hBMECs) derived from induced pluripotent stem cells (iPSC) from three healthy individuals and eight individuals with neurodegenerative disease. We systematically assessed protein and gene expression of BBB biomarkers, transendothelial resistance (TEER), permeability of Lucifer yellow, permeability of d-glucose, permeability of rhodamine 123, the efflux ratio of rhodamine 123, and P-gp inhibition using Tariquidar for confluent monolayers of human brain microvascular endothelial cell (hBMECs).

**Results:**

We provide evidence supporting the hypothesis that mutations associated with neurodegenerative disease can independently cause BBB dysfunction. These functional changes are not catastrophic since barrier breakdown would result in BBB impairment during development. Synergistic interactions between non-cell autonomous cerebrovascular dysfunction and the effects of gain-of-toxic function in neurons (e.g. toxic oligomers) are likely to increase disease burden through a positive feedback mechanism.

**Conclusions:**

These results suggest that the accumulation of defects in brain microvascular endothelial cells may ultimately lead to impairment of the BBB. Small changes in barrier function over time could lead to accumulated defects that result in positive feedback to unrelated central nervous system diseases.

**Electronic supplementary material:**

The online version of this article (10.1186/s12987-019-0139-4) contains supplementary material, which is available to authorized users.

## Background

Neurodegenerative diseases such as Alzheimer’s (AD), Parkinson’s (PD), Huntington’s (HD), and amyotrophic lateral sclerosis (ALS) impact 5.8 million American adults at an annual cost of about $250 billion in health care services, medication, and lost productivity [1, 2], similar to the annual cost of treating heart disease [3]. Establishing commonalities and differences amongst NDDs at the molecular, cellular, and organism levels is one current approach to elucidating disease mechanisms. While the precise mechanisms are unknown, neurodegenerative diseases (NDDs) share similar pathologies including: formation of intracellular and extracellular protein aggregates, gain of toxic function, activation of astrocytes and microglia, and upregulation of reactive oxygen species ultimately leading to neuronal cell death [4–9]. Another common feature of NDDs is the association with impairment of the blood–brain barrier (BBB), especially in late stage disease [10–25]. Although relatively few studies of the cerebrovasculature have focused on early stage neurodegenerative disease, evidence suggests that BBB impairment contributes to disease progression [6, 14, 18].

An emerging question in NDD research is whether mutations associated with NDDs cause non-cell autonomous dysfunction in other cell types. Support for this hypothesis comes from studies with patient-derived induced pluripotent stem cells (iPSCs). Neurons differentiated from iPSCs obtained from patients with AD, PD, HD, and ALS show hallmarks of the disease and gain of toxic function [26–39]. In the past 10 years, several studies have shown that mutations associated with NDDs are non-cell autonomous and cause dysfunction in other cell types. Amyloid-β accumulation has been observed in differentiated astrocytes from AD patients [40]. Differentiated HD astrocytes showed a significant increase in cytoplasmic vacuoles compared to controls [41]. Differentiated ALS astrocytes expressing the SOD1 or TDP-43 mutation, showed SOD-1 aggregates, decreased survival, and release of factors that are selectively toxic to motor neurons [42–44]. A recent study found that brain microvascular endothelial cells (BMECs) differentiated from four iPSC lines obtained from HD patients (60–109 CAG repeats in the *HTT* gene) showed increased angiogenic potential, decreased barrier function, and reduced efflux [45].

Establishing a causative relationship between NDD mutations and BBB impairment is challenging for two main reasons. Firstly, if mutations associated with NDDs lead to BBB impairment, then the effects are likely to be relatively small, otherwise symptoms would be observed during development. Secondly, since BBB impairment can occur in different processes that regulate normal BBB function, mutations associated with NDDs could result in impairment of the same process (“shared” phenotype) or different processes. To assess the role of selected mutations on BBB function, we derived human brain microvascular endothelial cells (dhBMECs) from 11 iPSC lines from three healthy individuals and eight individuals with NDDs: AD, PD, ALS, and HD. To capture a representative range of BBB function, we determined protein and gene expression of several BBB biomarkers, transendothelial electrical resistance (TEER), permeability (Lucifer yellow, d-glucose, and rhodamine 123), the efflux ratio of rhodamine 123, P-gp inhibition using Tariquidar, and oxidative stress. We show that seven out of eight dhBMECs derived from individuals with NDDs in an in vitro iPSC model show statistically significant impairment in transport/efflux systems and/or barrier function, providing support for the hypothesis that mutations associated with NDDs cause non-cell autonomous dysfunction of the BBB.

## Materials and methods

### Cell lines

Experiments were performed using 11 iPS cell lines (Table 1) from three healthy individuals and eight individuals with neurodegenerative disease.

**Table 1:** Description of iPS cell lines

Cell line	Disease	Mutation	Gender	Age isolated	Source tissue	Reprogramming method	Source
BC1	Healthy	N/A	M	36	Bone marrow CD34 + cell	Episomal, OCT4, SOX2, KLF4, c-MYC and LIN28	[46]
WT2	Healthy	N/A	F	56	Dermal fibroblast	Episomal, OCT4, SOX2, NANOG, LIN28, MYC, KLF4	[28]
iPS12	Healthy	N/A	F	Cord blood	Mesenchymal stromal cell	Episomal OCT4, SOX2, KLF4, L-MYC	Alstem
SODA4 V	ALS	SOD1 A4 V	F	57	Dermal fibroblast	Retroviral, Sox2, Oct4, Klf4, c-Myc	[47]
JH033	ALS	C9orf72 expansion	M	65	Dermal fibroblast	Retroviral, Sox2, Oct4, Klf4, c-Myc	[47]
SCNA1	PD	SCNA1 A53T	F	51	Fibroblast	Episomal	NINDS cell repository (NN0000052)
SCNAT	PD	SCNA1 triplication	F	55	Fibroblast	Episomal	NINDS cell repository (NN0000049)
AD10	AD	PSEN1 A246E	F	56	Dermal fibroblast	Episomal, OCT4, SOX2, NANOG, LIN28, MYC, KLF4	[28]
AD6	AD	PSEN1 A246E	F	56	Dermal fibroblast	Episomal, OCT4, SOX2, NANOG, LIN28, MYC, KLF4	[28]
HD 71	HD	HTT CAG:71	F	Unknown	Fibroblast	Episomal	NINDS cell repository (NN0003949)
HD 50	HD	HTT CAG:50	F	37	Fibroblast	Episomal, OCT4, SOX2, KLF4, L-MYC, LIN28	NINDS cell repository (NN0003930)

*ALS* amyotrophic lateral sclerosis, *PD* Parkinson’s disease, *AD* Alzheimer’s disease, *HD* Huntington’s disease

### Differentiation

iPSCs were maintained on six well plates (Grenier Bio-One, Monroe, NC) coated with Vitronectin in TeSR-E8 medium (Stem Cell Technologies, Vancouver, CDN) with daily media changes and passaged using StemPro Accutase (ThermoFisher Scientific, Waltham, MA). Cells were plated at a density to achieve an optimal confluence of 50–60% after 3 days to begin the differentiation. Cells were differentiated in UM/F-media for 5–6 days before being switched to endothelial cell media containing retinoic acid (RA, 10 μM, Millipore Sigma, St. Louis, MO) and 2 ng mL^−1^ bFGF (R&D Systems) for 2 days, as described previously [48]. Differentiations were evaluated based on the following characteristics. On day 2, the cells reached complete confluence providing the optimal density of neural precursor cells and endothelial cells through the differentiation. On day 3, macroscopic neural tracts, which were visible to the naked eye, formed a network pattern throughout the well. On day 5, areas of the monolayer became more optically transparent and under the microscope the cells in these regions had a cobblestone-like morphology. When these regions expanded to fill most of the area between the neural tracts, the cells were switched to endothelial cell medium for subculture; usually on day 6. All of the cell lines studied here progressed through the differentiation in the same way, comparable to the BC1 line [49], with the exception of the WT2 iPSCs. Differentiation of the WT2 line progressed rapidly between days 3–5 and were switched to endothelial cell medium on day 5. After 2 days in endothelial cell medium, the neural tracts receded and the endothelial cells continued to proliferate and mature.

Differentiated cells were plated on transwell inserts or tissue culture plates coated with collagen IV and fibronectin at a density of 10^6^ cells mL^−1^ (tissue culture plates) and 5 × 10^6^ cells mL^−1^ (transwell inserts), and all experiments were performed 2 days following sub-culture unless otherwise noted.

### Immunofluorescence

Briefly, dhBMECs were sub-cultured onto glass-bottomed dishes coated overnight with 50 mg/mL collagen IV and fibronectin. After 48 h, cells were fixed using 3.7% paraformaldehyde, and were then permeabilized using 0.1% Triton-X, blocked using 10% donkey serum in PBS azide, and stained using primary antibodies for claudin-5 (ThermoFisher Scientific, 35-2500), ZO-1 (ThermoFisher Scientific, 40-2200), occludin (ThermoFisher Scientific, 33-1500), and GLUT1 (Abcam, Cambridge, UK, ab115730), followed by incubation with Alexa Fluor conjugated secondary antibodies (ThermoFisher Scientific). Stained samples were imaged on a Nikon TiE microscope using a 60× oil immersion objective.

### qRT-PCR

For qRT-PCR, dhBMECs were sub-cultured onto 6-well tissue culture plates and lysed using cells-to-CT kit (ThermoFisher Scientific). Cell lysate was prepared using the TaqMan Gene Expression Kit (ThermoFisher Scientific), using TaqMan Probes (ThermoFisher Scientific). qPCR was performed using a StepOnePlus Real-Time PCR system (ThermoFisher Scientific). Fold change was analyzed using the comparative CT method (ΔΔC_t_) normalizing to *ACTB* and *GAPDH* expression with BC1-dhBMECs used as a reference. All experiments were performed over three separate differentiations (N = 3) with three technical replicates for each differentiation.

### Western blot

For Western blotting, dhBMECs were sub-cultured onto T-25 tissue culture plates and lysed with RIPA buffer with protease inhibitor cocktail (Millipore Sigma). Lysate was reduced and run on 4–15% pre-cast polyacrylamide gels (Bio-Rad, Hercules, CA) and transferred to nitrocellulose membranes (Bio-Rad). The membranes were then blocked and stained in 5% fat-free skim milk in TBST with 0.05% TWEEN-20 using primary antibodies for claudin-5 (ThermoFisher Scientific, 35-2500), occludin (ThermoFisher Scientific, 33-1500), P-gp (Millipore Sigma, P7965), and ZO-1 (ThermoFisher Scientific, 40-2200). Blots were developed and imaged using Bio-Rad molecular imager ChemiDoc XRS+. Bands were normalized to the intensity of the β-actin band and compared to the BC1-dhBMEC lane. All experiments were performed over three independent differentiations (N = 3) with duplicate technical replicates for each differentiation.

### TEER and permeability

Transendothelial electrical resistance and permeability measurements were performed on dhBMEC monolayers sub-cultured onto 0.33 cm^2^ polyester Transwell membranes (0.4 µm pore size, Corning, Corning, NY) with transport buffer (distilled water with 0.12 M NaCl, 25 mM NaHCO_3_, 3 mM KCl, 2 mM MgSO_4_, 2 mM CaCl_2_, 0.4 mM K_2_HPO_4_, 1 mM HEPES, and 1% human platelet poor derived serum) in both apical and basolateral chambers. TEER was measured daily for 1 week using an EVOHM2 with STX2 probes (World Precision Instruments, Sarasota, Fl). TEER measurements were performed over four separate differentiations (N = 4) with 5–11 technical replicates for each differentiation.

Permeability measurements were performed with 100 µM Lucifer yellow, 10 µM rhodamine 123, and 25 mM d-glucose. The apical-to-basolateral permeability of 100 µM Lucifer yellow (ThermoFisher Scientific) was measured after 60 and 90 min. At each time point the apical well was removed from the basolateral well [48]. The permeability of 10 µM rhodamine 123 (ThermoFisher Scientific) was measured in both apical-to-basolateral and basolateral-to-apical directions at 30 and 60 min. Apical-to-basolateral measurements was performed in the same way as for Lucifer yellow with the apical well being removed at 30 and 60 min. Basolateral-to-apical measurements were performed at 30 and 60 min by removing the buffer contained in the apical chamber and diluting for later measurement of the solute. For inhibition experiments, dhBMEC monolayers in the transwells were incubated for 10 min in 2 μM Tariquidar in endothelial cell medium with RA, prior to replacing with fresh transport buffer containing rhodamine and 2 μM Tariquidar. All permeability measurements were performed for three separate differentiations (N = 3) with triplicate technical replicates for each differentiation.

The amount of Lucifer yellow or rhodamine 123 in the basolateral chamber or the diluted apical chamber was measured using a plate reader (BioTek™ Synergy™ H4). Calibration curves were obtained from serial dilutions. The apical-to-basolateral permeability of 25 mM d-glucose (Millipore Sigma) was measured in transport buffer without serum at 5, 10, 15, and 30 min. The amount of d-glucose transported across the monolayer was measured using a glucose colorimetric detection kit (ThermoFisher Scientific) and plate reader. Each permeability experiment was run at the same time as a serial dilution of standards spanning at least five orders of magnitude, starting at the input concentration. The apparent permeability was determined as previously reported [48]. The time points for the permeability measurements were previously optimized to account for differences in permeability, the detection range of the plate reader, and to be within the linear concentration range in the basolateral chamber [50].

### ROS assay

Reactive oxygen species (ROS) assays were performed on dhBMECs sub-cultured onto a 96 well plate. Oxidative stress was induced using 5 or 50 mM menadione in growth medium, and incubation growth medium containing 0.5% ethanol was used as control. CellROX green (ThermoFisher Scientific) was added to the medium to assess the production of ROS, and the plate was imaged on a plate reader to determine the amount of accumulated ROS after 30 and 60 min. The control well containing medium, ethanol, and cellROX was used as a baseline for all conditions. The response to oxidative stress is reported as percent increase compared to the control. ROS measurements were performed over three separate differentiations (N = 3) with duplicate technical replicates for each differentiation.

### Statistical analysis

The statistical significance between the individual disease lines and the control lines were determined using a nested ANOVA test where the disease was the main group, with each cell line as a subgroup. Additionally, a paired t-test was used to determine if there were any global differences between the three healthy control lines (BC1, WT2, and iPS12) and all of the disease lines (JH033, SODA4V, SCNA1, SNAT, AD6, AD10, HD50, and HD71).

A cluster analysis was carried out in R (v3.5.0) to assess whether individual samples clustered by disease status. Data were clustered on the functional assay measurements for each sample: d-glucose, ER, LY permeability, and TEER using the R package flipCluster (v1.1.0), which allows for partial data, such as we have in this case where there are missing measures in some samples. Each sample was assigned to one of two clusters. Clustering was assessed by determining the number of samples from each cell line that clustered into each of the two clusters. Code for this analysis can be found at: https://github.com/ShanEllis/NDD.

## Results

To assess the role of mutations associated with neurodegenerative disease (NDD) in inducing BBB impairment, we selected 11 cell lines: two lines for each NDD and three healthy controls (Table 1). Disease lines were chosen to exemplify common mutations associated with NDDs. In ALS, SOD1 and C9orf72 expansion are the two most common mutations [51]. In PD, SCNA1 mutations are common in familial disease, and two of the more common permutations (SCNA1 A53T and SCNA1 triplication) were selected [52]. PSEN1 mutations are common in familial AD, and we selected two lines with the same mutation from individuals of both genders and the same age [28]. HD lines were chosen with short and moderate CAG expansions to complement existing work [45]. Differentiation of dhBMECs was performed following our previously published protocol [48, 49, 53]. Differentiations of the NDD lines were indistinguishable from the healthy lines, showing the appearance of neural tracts around day 3, and the appearance of a well-defined cobblestone morphology characteristic of endothelial cells on days 4–6, clearing up by day 8 (Additional file 1: Figure S1).

Blood–brain barrier function was assessed from protein and gene expression levels of selected biomarkers, transendothelial electrical resistance (TEER), permeability (Lucifer yellow, d-glucose, and rhodamine 123), the efflux ratio of rhodamine 123, P-gp inhibition using Tariquidar, and oxidative stress. The number of cell lines, molecular characterizations, and functional measurements were selected to provide a representative range of common mutations and assessment of possible impairment processes common across neurodegenerative diseases. In analyzing barrier function we considered differences between individual disease lines and the healthy controls as well as grouped differences between the disease lines and the controls.

### Protein and gene expression

**Fig. 1:**
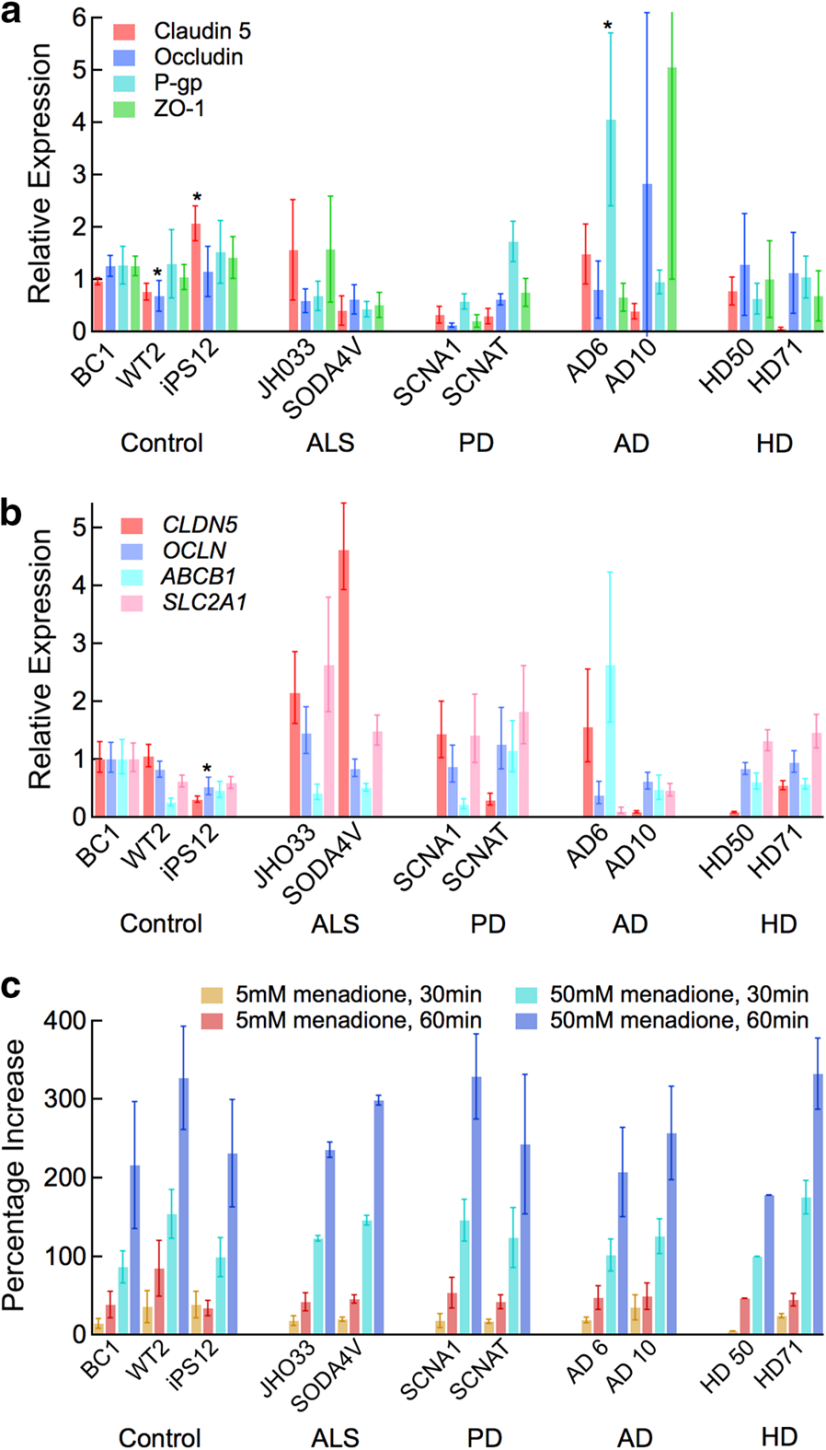
Protein and gene expression in dhBMECs from healthy individuals and individuals with NDD patients. **a** Protein expression from Western blots for claudin-5, occludin, P-gp, and ZO-1 for all 11 cell lines. Data were obtained from two technical replicates for each of three independent differentiations (N = 3). Results were normalized by first correcting for protein concentration based on β-actin loading control, and then normalized to the first technical replicate of BC1 run on each gel. Selected bands are provided in Additional file 1: Figure S2. **b** mRNA expression assessed using qRT-PCR for CLDN5, OCLN, ABCB1 (P-gp), and SLC2A1 (GLUT1) for all 11 cell lines. Expression levels are normalized to the mean expression level for that gene in the BC1 dhBMEC line after normalization to the housekeeping genes *BACT* and *GPADH*. Data were obtained from three technical replicates for each of three differentiations (N = 3). **c** Percent increase in amount of reactive oxygen species in response to stress induced by exposure to 5 and 50 mM menadione for 30 and 60 min. Data were obtained from two technical replicates for each of three differentiations (N = 3). Error bars represent mean ± SE. Statistical analysis was performed using nested ANOVA comparing to the three healthy controls. *p < 0.05

dhBMECs from healthy and disease lines all expressed proteins (claudin-5, occludin, ZO-1, and P-gp) associated with the BBB (Fig. 1a). Western blots are shown in Additional file 1: Figure S2 and the expression of individual proteins across all disease lines in Additional file 1: Figure S3. Comparison of expression levels by disease are shown in Additional file 1: Figure S4. Protein expression showed little variation within biological replicates, with the exception of the AD cell lines. The expression levels and variation between biological replicates for the BC1 cell line were similar to our previously reported results [48, 53]. The largest variability in protein expression was within the three healthy controls. Compared to the BC1 and iPS12 lines, the WT2 lines showed significantly lower occludin expression (p < 0.05), and the iPS12 line showed significantly higher claudin-5 expression (p < 0.05) than the BC1 and WT2 lines. This variability within the heathy controls is important to capture as it suggests that variation across individuals is significant and likely accounts for some of the variability across the disease lines. The only line that showed a significant difference in protein expression from the three healthy lines was the AD6 line, which showed increased expression of P-gp (p < 0.05). When the results from all of the disease lines were combined and compared to the combined healthy control lines, claudin-5 expression was significantly lower (p < 0.05), however, no individual diseased line maintained this significant difference.

Similar to the results from Western blots, the variability in the expression of four genes associated with the BBB (CLDN5, OCLN, ABCB1, and SLC2A1) was relatively large among the healthy controls (Fig. 1b). Grouping all disease lines, the only significant difference compared to the healthy lines was a decrease in the level of occludin expression (p < 0.05). The relative protein and gene expression levels (Fig. 1b) were normalized to the BC1 line, however, all statistical tests were performed in comparison to all three healthy cell lines. When results were normalized compared to one of the other healthy controls the distribution changed slightly, more so when the iPS12 line was used as a reference, but the statistical trends were the same (Additional file 1: Figure S5).

### Stress response

A common feature of neurons in NDDs is their poor response to stress [54], resulting in increased accumulation of reactive oxygen species (ROS). For example, neurons differentiated from iPSCs from individuals with NDDs showed increased generation of ROS compared to healthy controls [7, 31, 55]. We measured the levels of ROS in the dhBMEC lines following incubation with menadione but found no statistical difference in ROS levels in cells derived from individuals with NDDs compared to healthy controls (Fig. 1c). Menadione generates ROS through futile redox cycling as a stress response, and is commonly used at low concentrations to recapitulate oxidative stress in vitro [56].

### Immunofluorescence images

**Fig. 2:**
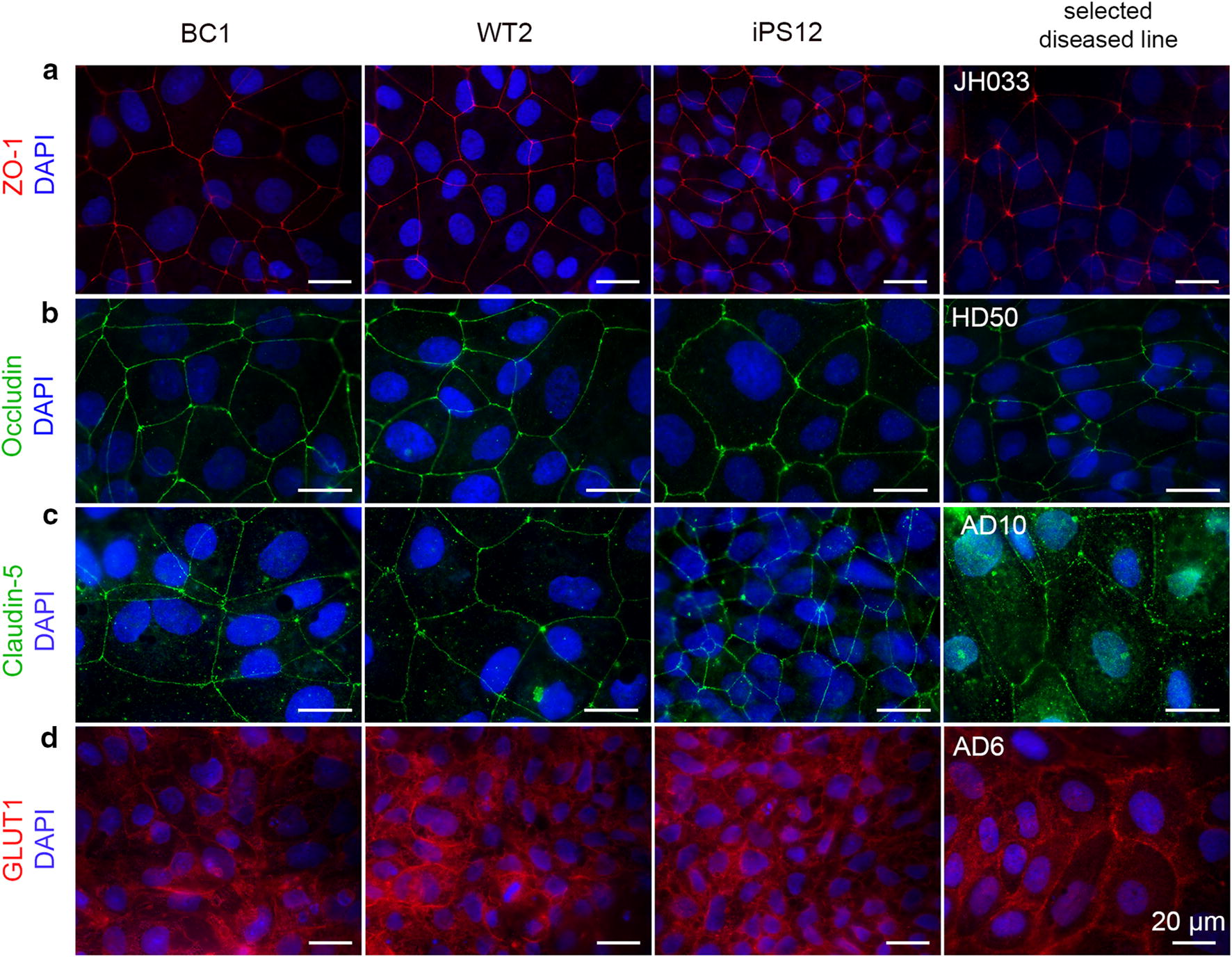
Protein localization in dhBMEC monolayers derived from healthy individuals and individuals with NDD. The first column show images from the healthy controls WT2. The last column shows images from a selected NDD hBMEC line highlighting changes in protein localization if any were evident. The rows correspond to: **a** ZO-1, **b** occludin, **c** claudin-5, and **d** the GLUT1 transporter. The tight junction markers were localized at junctions in all cell lines. The GLUT1 transporter showed blanket staining over the cell membrane for all monolayers. Additional stains can be found in Additional file 1: Figures S6–S9

Immunofluorescence imaging was performed on confluent monolayers of dhBMECs sub-cultured on collagen IV and fibronectin coated glass to visualize localization of tight junction proteins (ZO-1, occludin, claudin-5) as well as glucose transporter 1 (GLUT1). Representative images for confluent monolayers of dhBMECs are presented here (Fig. 2). Additional images can be found in Additional file 1: Figures S6–S9. All dhBMEC lines showed continuous ZO-1 junctional networks, although some disease lines, notably the JH033 line, showed increased staining at the triple points (Fig. 2a, Additional file 1: Figure S6). Quantitative analysis of the ZO-1 stains indicated that there was no significant change in the average cell area in the monolayers (Additional file 1: Figure S10). Occludin staining similarly showed continuous junctions, and no apparent changes in occludin staining were observed across the disease lines (Fig. 2b, Additional file 1: Figure S7). Claudin-5 staining was localized to the junctions in the healthy cells and in most of the disease lines, although junctions appeared slightly less continuous in the AD10 line (Fig. 2c, Additional file 1: Figure S8). Analysis of the claudin-5 images revealed that the SCNA1, AD6, and AD10 lines exhibited a higher fraction of frayed junctions than the controls (Additional file 1: Figure S10). Since Western blots of GLUT1 are complicated by the large molecular weight range associated with the level of glycosylation [57], immunohistochemistry provides a qualitative alternative to ensure protein expression and localization of this important transporter. GLUT1 showed global expression in the monolayers, with localization on the plasma membrane (Fig. 2d, Additional file 1: Figure S9).

### Barrier function

Barrier function was assessed from measurement of transendothelial electrical resistance (TEER) and solute permeability. Permeability was measured for Lucifer yellow (MW 444 Da), a small cationic molecule widely used to assess barrier integrity, rhodamine 123, a substrate of several efflux pumps including the P-gp efflux pump, and d-glucose, a substrate of the GLUT1 transporter.

**Fig. 3:**
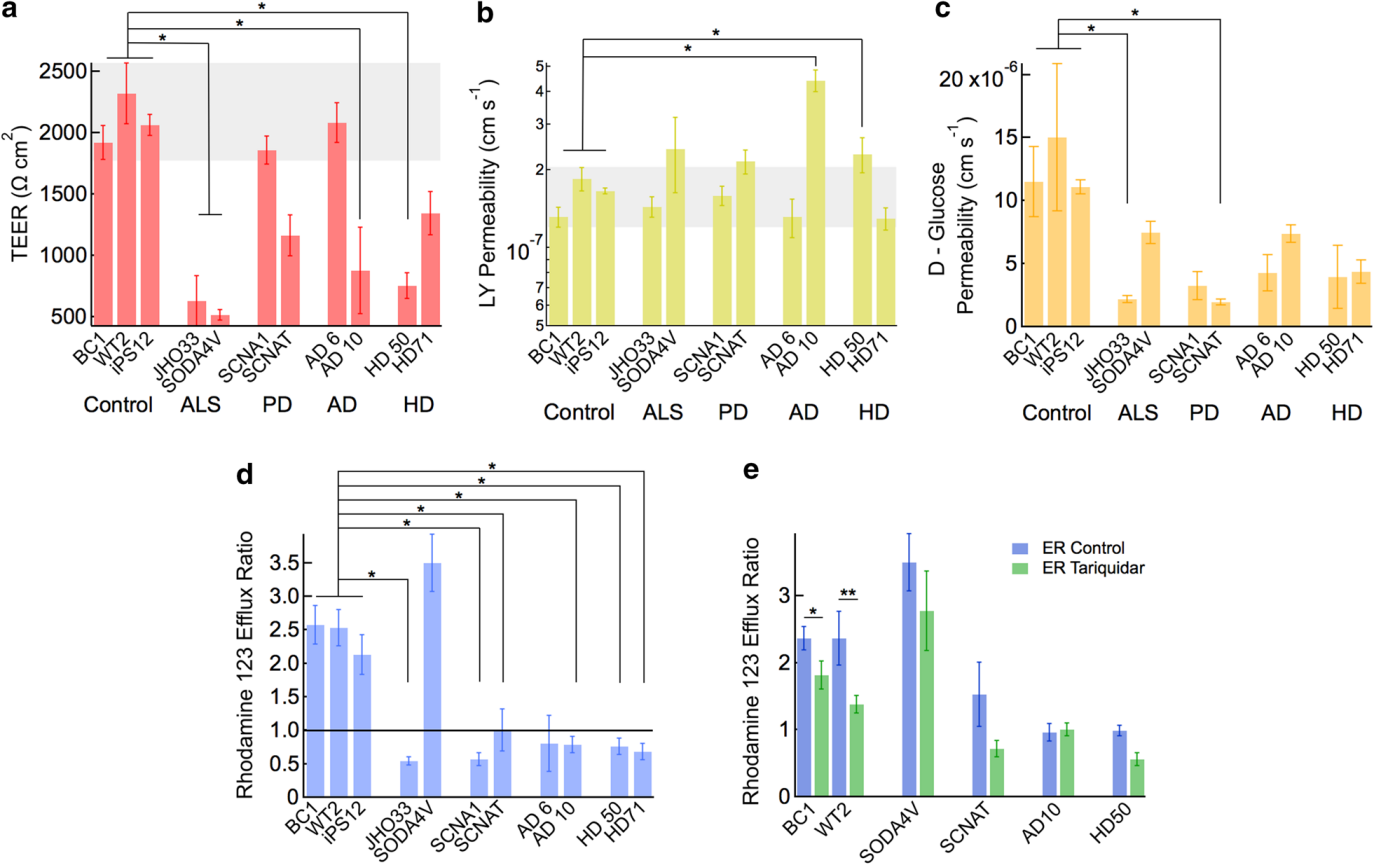
Barrier function of dhBMEC monolayers derived from healthy individuals and individuals with NDD. **a** Transendothelial electrical resistance (TEER) showing significant decreases in 4/8 disease lines compared to healthy controls. **b** Permeability of Lucifer yellow showing that normal barrier function is maintained in all disease lines except the AD10 line. **c** d-Glucose permeability showing decreased transport in the NDD lines. **d** Efflux ratio for rhodamine 123, determined from the ratio of the basolateral-to-apical and apical-to-basolateral permeabilities. **e** Efflux ratio for rhodamine 123 with and without Tariquidar inhibition for six selected dhBMEC cell lines. The grey bands correspond to the lowest and highest values of the SE for the healthy controls. Error bars represent mean ± SE. Statistical analysis was performed using nested ANOVA comparing to the three healthy controls. *p < 0.05. All experiments were performed in triplicate for each of three different differentiations

Transendothelial electrical resistance values for the healthy controls were between 1800 and 2500 Ω cm^2^, consistent with previous studies of dhBMECs from healthy individuals [45, 48, 58], and in the range of physiological values in animal models (1500–8000 Ω cm^2^) [59–63]. TEER values for the two ALS lines, and one each of the PD, AD, and HD lines were statistically significantly lower than the healthy controls. For these cell lines, TEER values were in the range 500–1000 Ω cm^2^, below physiological values but relatively high compared to values measured in many primary or immortalized lines (Fig. 3a).

The Lucifer yellow permeability for healthy dhBMEC monolayers was 1–2 × 10^−7^ cm s^−1^ (Fig. 3b), similar to values reported in a rat model [64]. Values below 1 × 10^−6^ cm s^−1^ are considered to be consistent with normal barrier function and restricted paracellular transport [65]. All disease lines exhibited permeabilities similar to the controls except the AD10 line which (4.42 ± 0.42 × 10^−7^ cm s^−1^; p < 0.05) and the HD50 line (2.3 ± 0.35 × 10^−7^ cm s^−1^; p < 0.05) indicating a small but statistically significant decrease in barrier function.

The permeability of d-glucose reflects the function of the glucose transporter GLUT1. For the healthy dhBMEC lines, the glucose permeability was 1.1–1.5 × 10^−5^ cm s^−1^ (Fig. 3c), similar to values reported in the literature [66], while the disease lines had glucose permeabilities of 0.19–0.75 × 10^−5^ cm s^−1^. When individual NDD lines were compared to grouped healthy controls, only the SCNAT and JH033 lines were statistically lower (p < 0.05).

The apical-to-basolateral permeability of rhodamine 123 was 0.8–3 × 10^−7^ cm s^−1^ for the healthy lines, and 0.4–5 × 10^−7^ cm s^−1^ for the disease lines (Additional file 1: Figure S11). Rhodamine 123 is a substrate of several efflux pumps, including the P-pg pump. The effectiveness of efflux is measured as the efflux ratio of the basolateral-to-apical and apical-to-basolateral permeabilities. Since the P-gp pumps are polarized to the apical surface, for solutes that are P-gp substrates the efflux ratio is greater than 1.0. All three healthy control lines showed an efflux ratio of 2–4, consistent with previous reports and indicative of active efflux transporters polarized to the apical membrane [48]. In contrast, all of the disease lines, except SOD4AV, showed an efflux ratio ≤ 1 (p < 0.05), suggesting reduced functionality or incorrect polarization of efflux transporters (Fig. 3d).

Following inhibition of P-gp with Tariquidar, a P-gp inhibitor, the efflux ratio of rhodamine 123 was reduced, indicative of partial inhibition (Fig. 3e). This reduction in efflux ratio was seen across many of the lines tested here, but was only significant in the healthy controls (p < 0.05).

### Cluster analysis of BBB impairment

**Fig. 4:**
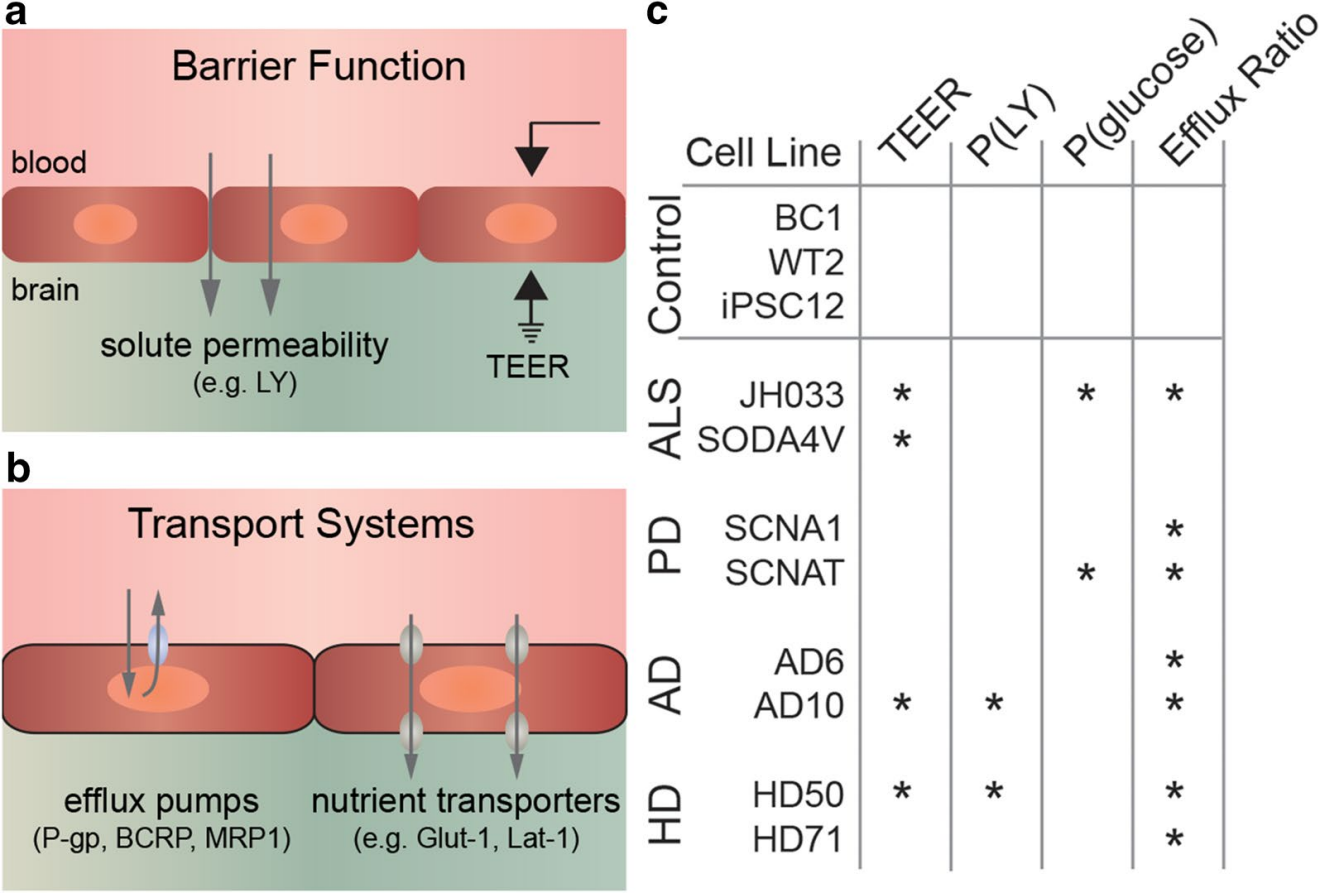
Summary of grouped characterization of barrier function and transport systems for healthy lines (BC1, WT2, and iPS12) and disease lines (JH033, SODA4V, SCNA1, SCNAT, AD6, AD10, HD50, HD71). **a** Schematic illustration of barrier function. **b** Schematic illustration of transport systems. **c** Summary of impairment in TEER, P(LY), P(glucose), and efflux ratio for disease lines with respect to grouped healthy controls. TEER measurements for healthy lines (N = 13 independent differentiations), and disease lines (N = 30 independent differentiations). Lucifer yellow permeability for healthy lines (N = 9, 3 independent differentiations), and disease lines (N = 24 independent differentiations). Glucose permeability for healthy lines (N = 10 independent differentiations), and disease lines (N = 26 independent differentiations). Efflux ratio for rhodamine 123 permeability for healthy lines (N = 14 independent differentiations), and disease lines (N = 33 independent differentiations). Statistical analysis was performed using nested ANOVA comparing to the three healthy controls. *p < 0.05

**Fig. 5:**
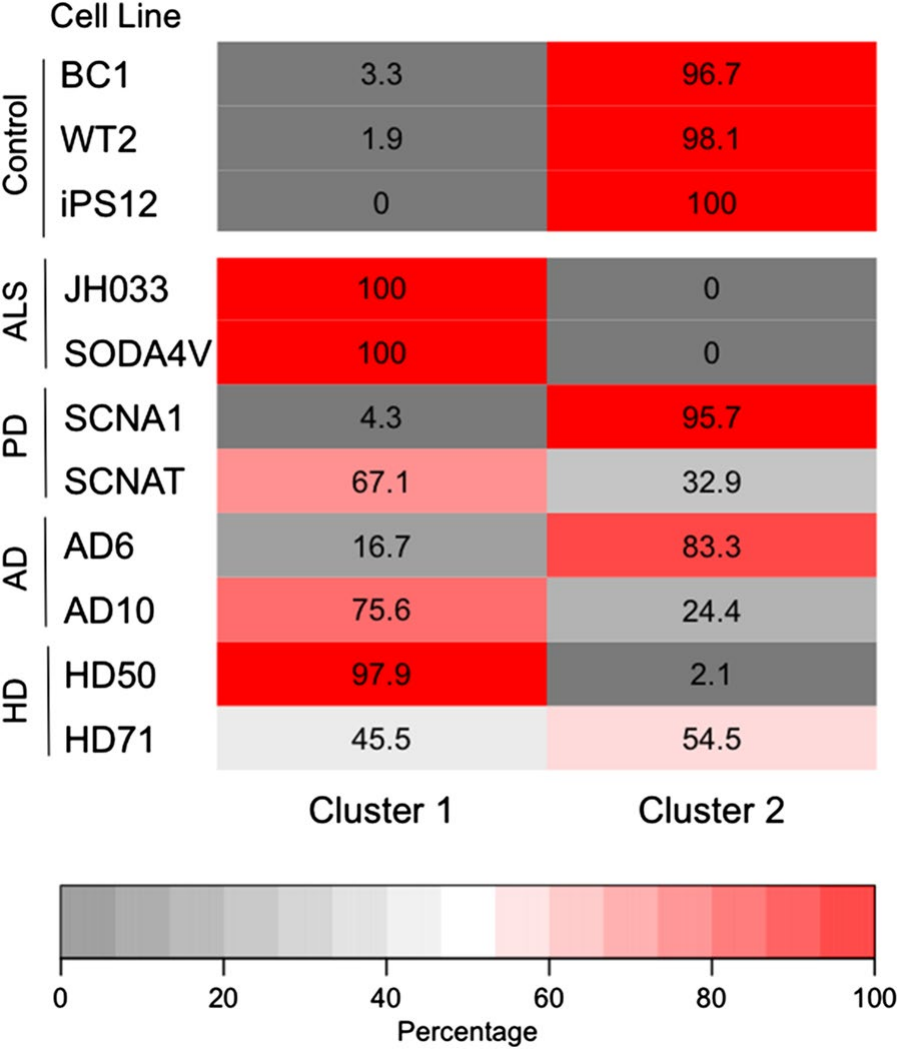
Cluster analysis demonstrates a phenotypical difference between healthy controls to NDD lines. This heatmap demonstrates the percentage of measurements in from each cell line within each cluster. Analysis is based on 599 measurements (TEER, Lucifer yellow permeability, glucose permeability, and Rhodamine efflux ratio): BC1 (N = 41), WT2 (N = 64), iPS12 (N = 53), JH033 (N = 52), SODA4V (N = 60), SCNA1 (N = 56), SCNAT (N = 81), AD6 (N = 69), AD10 (N = 52), HD50 (N = 57), and HD71 (N = 64). Rows are sorted by cell line. Colors correspond to strength of representation within each cluster where gray shows limited representation of that cell line within that cluster and red represents that most samples within that cell line fall within that cluster

The four metrics of barrier function [TEER, P(LY), P(glucose), and efflux ratio] reveal dysfunction of at least one metric in all disease lines (Fig. 4). A decrease in the efflux ratio was the most common dysfunction, present in 7/8 disease lines. A comparison of barrier function based on grouped disease lines is provided in Additional file 1: Figure S12. To globally assess the barrier function of dhBMEC monolayers between cell lines from individuals with NDDs and healthy controls, we performed a cluster analysis (Fig. 5). The analysis was based on a total of 599 measurements and included all biological and technical replicates from TEER, and all biological replicates from Lucifer yellow permeability, glucose permeability, and rhodamine efflux ratio. Two clusters were generated to determine if there were any patterns or clustering of dhBMEC barrier function measurements across all NDD lines. Overall, 98.4% of the measurements from the healthy lines appeared in Cluster 2. A majority of the measurements from the disease lines appeared in Cluster 1 (61.6%); however, 38.4% of the measurements clustered with the healthy samples in Cluster 1. When categorized by cell line, it is evident that certain cell lines (namely SCNA1 and AD6) tend to cluster more closely with the healthy cell lines. Specifically, the NDD dhBMEC lines that had TEER values similar to the healthy controls tended to shift toward the cluster with the healthy lines (Cluster 2). This analysis further demonstrates a subtle phenotype shift in the dhBMECs derived from NDD lines compared to the healthy controls.

## Discussion

**Fig. 6:**
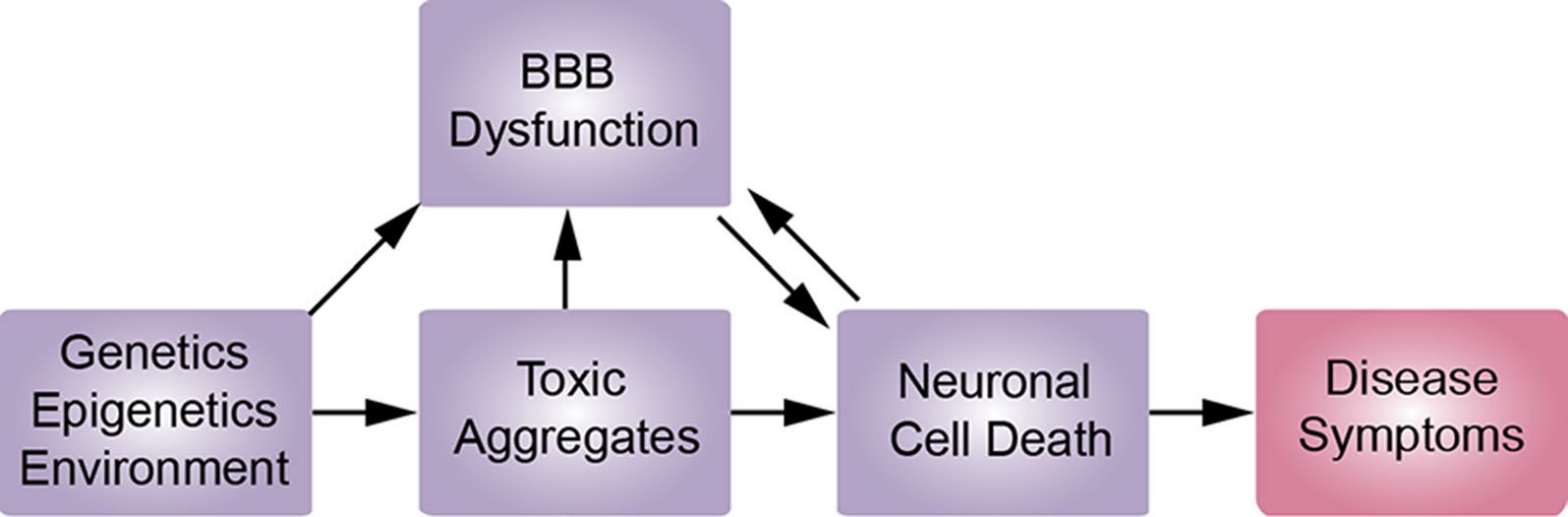
The role of BBB dysfunction in the progression of neurodegenerative disease. BBB dysfunction is associated with many late stage neurodegenerative diseases and is thought to be a consequence of accumulation of toxic aggregates and neuronal cell death. BBB dysfunction can lead to entry of blood components and compromised nutrient transport, providing positive feedback for disease progression. We propose that mutations associated with neurodegenerative diseases can independently lead to accumulation of defects in the blood–brain barrier, ultimately resulting in increased paracellular permeability and/or compromised transport systems (e.g. efflux of non-essential molecules or decreased nutrient transport). Prior to the onset of measurable changes in barrier function, accumulation of defects in the BBB may occur independently of or in parallel with gain of toxic function in neurons, or other cell types in the brain

Blood–brain barrier dysfunction is considered a hallmark of NDDs [10–25], however, most of our understanding of the relationship between BBB impairment and NDDs comes from late stage disease. Very little is known about whether BBB impairment contributes to, or is a consequence of, disease progression [13, 67]. One explanation is that mutations associated with NDDs are acquired by brain microvascular endothelial cells, leading to BBB impairment as an independent or co-occurring pathology (Fig. 6). Here we test this hypothesis by studying the barrier function of confluent monolayers of human brain microvascular endothelial cells derived from iPSCs from healthy individuals and individuals with four different NDDs.

Functional BBB impairment can be broadly classified as either changes in paracellular transport (e.g. increased paracellular permeability, immune cell trafficking, etc.) or dysfunction of transport systems (e.g. dysfunction of efflux or nutrient transport) (Fig. 4a, b). Here we assess impairment of paracellular transport (TEER, Lucifer yellow permeability) and transcellular transport (efflux ratio for rhodamine 123, P-gp inhibition, glucose permeability).

First, we consider paracellular transport. Although the decrease in TEER values for dhBMEC monolayers derived from NDD lines is relatively large, in all cases the TEER values are in excess of 500 Ω cm^2^. Previous studies have shown that dhBMECs obtained using the standard 2-step differentiation show an increase in sodium fluorescein permeability for TEER values ≤ 500 Ω cm^2^, implying that barrier function is maintained above this threshold [68]. This is confirmed by the permeability of Lucifer yellow, which was only larger than the grouped controls for the AD10 and HD50 lines. Similarly, we observed no systematic changes in expression of tight junction markers, however, the AD10 line was one of three lines with an increase in fraction of frayed claudin-5 junctions. Taken together, these results suggest that dhBMECs derived from patients with NDDs result in very little change in barrier function associated with paracellular transport. This conclusion is consistent with pathophysiological evidence: if mutations associated with NDDs and acquired by brain microvascular endothelial cells resulted in barrier breakdown then cerebrovascular pathologies would be observed during development. However, small changes in regulation of paracellular transport could accumulate and lead to breakdown over time. For example, there is evidence that paracellular transport is increased in the spinal chord in an ALS mouse model before the onset of symptoms [18], although pre-symptomatic disruption is not observed in larger rat models [69].

A recent study of dhBMECs derived from four individuals with HD (CAG repeats of 60, 66, 71, and 109) only showed a decrease in TEER below physiological values (< 1500 Ω cm^2^) for CAG repeats of 71 and 109 [45]. Similar to the results reported here, dhBMECs from individuals with HD showed no systematic changes in the levels of claudin-5 or occludin expression compared to dhBMECs from healthy individuals. Functional permeability measurements were not reported in this study. Here we show low TEER values (500–1500 Ω cm^2^) for lines derived from patients with 50 and 71 CAG repeats, and no observable differences in Lucifer yellow permeability. Together, these results suggest small decreases in the tightness of the junctions (TEER) but no functional differences in the permeability of a small molecule (Lucifer yellow).

Next, we consider the function of transport systems in dhBMEC monolayers. First, we consider glucose transport and then efflux transport. While the NDD lines show lower average glucose permeability than the controls, only the SCNAT and JH033 lines were statistically significant. Decreased GLUT1 expression and function has been reported in AD, and the glucose concentration in CSF has been proposed as a possible early indicator of increased risk of AD [70–72]. Nutrient transport is critical for normal neuronal function and changes in nutrient transport have been found to be disease inducing. For example, in GLUT1 deficiency syndrome, attenuation of d-glucose transport into the brain results in early onset encephalopathy and seizures which can be mediated by a ketogenic diet [73]. In autism spectrum disorder, mutations in large amino acid transporter 1 (LAT1) result in deficiencies in branched chain amino acids in the brain that result in neurological anomalies [74].

The role of efflux transporters in the cerebrovasculature is critical in restricting access to the brain and maintenance of brain homeostasis. Seven of the eight NDD lines show significantly lower efflux ratio compared to the healthy controls. Numerous studies have reported decreased expression of the P-gp efflux pump in tissue samples from AD patients and in mouse models of AD [13, 15, 67, 72, 75–77]. In mouse models of AD, deficiencies in P-gp have been shown to exacerbate disease symptoms [67]. It is well known that P-gp dysfunction plays a role in late stage disease progression of AD by reducing the ability to clear amyloid β from the brain [78]. Polymorphism in P-gp appears to be a risk factor for PD [79, 80], where one of the main genetic mutations associated with the development of PD is a P-gp mutation. The dysfunction of P-gp efflux can cause multiple downstream effects, including the increased penetration of blood components and other components in circulation [15, 23, 78]. dhBMECs derived from individuals with HD showed increased rhodamine 123 uptake compared to dhBMECs from healthy controls, suggesting possible P-gp dysfunction [45]; rhodamine 123 uptake is often used as a proxy for efflux pump function.

The decreased efflux ratio in 7/8 disease lines combined with the comparable level of P-gp protein and transcript expression suggests that P-gp is present in the cell but unable to efflux known substrates. Possible explanations include: lack of appropriate trafficking to the membrane, fast recycling, or lack of polarization to the apical surface. Understanding how P-gp localization and trafficking may be altered in NDD could provide insight into possible therapeutic targets.

In summary, we have characterized paracellular transport (TEER, Lucifer yellow permeability) and transcellular transport (efflux ratio for rhodamine 123, P-gp inhibition, glucose permeability) of NDD lines compared to healthy controls. Overall, we summarize the results for individual cell lines as follows. (1) 4 of 8 NDD lines show a decrease in TEER, which can’t be explained by changes in protein or gene expression (Fig. 1), tight junctional localization (Fig. 2 and Additional file 1: Figures S6–S8), or tight junction continuity (Additional file 1: Figure S10). Functionally, this could be due to a decrease in the number of pinning points associated with the heterotypic interaction between extracellular claudin-5 domains, or due to a decrease in the area of cell–cell overlap. (2) Only 2 of 8 NDD lines show significantly higher permeability in small molecule permeability (Lucifer yellow). This increase is small, however, with the largest change being a factor of 2 (AD10). This suggest that any changes in tight junction ultrastructure are not sufficient to allow a large increase in paracellular transport of small molecules. (3) 2 of 8 NDDs show a decrease in glucose permeability. (4) 7 of 8 NDDs show a decrease in efflux ratio for rhodamine 123, suggesting dysfunction of the P-gp transport system compromising the ability of the cerebrovasculature to restrict access of non-essential molecules to the brain.

These results provide support for the hypothesis that mutations associated with NDDs could lead to BBB impairment, and provide rationale for further studies. In addition, these results suggest that it is unlikely that there is a “shared” phenotype, highlighting the need for comprehensive assessment of barrier function in future studies. Finally, the results indicate that that the dhBMEC model is sufficient to capture subtle changes in phenotype associated with BBB impairment.

## Conclusions

Taken together, these results suggest that the accumulation of defects in brain microvascular endothelial cells may, in many cases, ultimately lead to BBB impairment. Small changes in barrier function over time could lead to accumulated defects that provide positive feedback to unrelated CNS diseases, resulting in increased dysfunction and symptoms of cerebrovascular disease. Furthermore, these results, although in a limited number of cell lines, suggest that cerebrovascular dysfunction may occur independent of neurodegeneration and may be common to neurodegenerative diseases. This correlation also suggests potential new opportunities for therapeutic intervention and diagnosis.

## Additional file

**Additional file 1: Figure S1.** Phase images of differentiation of iPSCs. **Figure S2.** Western blots. **Figure S3.** Expression of individual proteins and genes across all disease lines. **Figure S4.** Protein and gene expression by disease. **Figure S5.** Gene expression normalized to WT2 and iPS12 lines. **Figure S6.** Additional stains: ZO-1. **Figure S7.** Additional stains: occludin. **Figure S8.** Additional stains: claudin-5. **Figure S9.** Additional stains: Glut-1. **Figure S10.** Cell area and fraction of frayed junctions. **Figure S11.** Rhodamine 123 permeability. **Figure S12.** Transport dysfunction by disease.

## Data Availability

Not applicable.

## Abbreviations

ABCB1: ATP binding cassette subfamily B member 1 (P-gp)
ACTB: beta-actin
AD: Alzheimer’s disease
ALS: amyotrophic lateral sclerosis
BBB: blood–brain barrier
bFGF: basic fibroblast growth factor
BMEC: brain microvascular endothelial cell
CAG: trinucleotide repeat expansion located in the first exon of the HD gene
CLDN5: claudin-5
CNS: central nervous system
DAPI: 4′,6-diamidino-2-phenylindole fluorescent stain
dhBMECs: differentiated human brain microvascular endothelial cells
EC: endothelial cell
ER: efflux ratio
GAPDH: glyceraldehyde-3-phosphate dehydrogenase
GLUT1: glucose transporter 1
hBMECs: human brain microvascular endothelial cells
HD: Huntington’s disease
HTT: Huntingtin protein
iPSC: induced pluripotent stem cell
LY: Lucifer yellow
MW: molecular weight
NDDs: neurodegenerative diseases
OCLN: occludin
P_app_: apparent permeability
PBS: phosphate buffered saline
PD: Parkinson’s disease
P-gp: p-glycoprotein
PSEN1: presenilin-1
qPCR: quantitative polymerase chain reaction
ROCK: rho-associated protein kinase
ROS: reactive oxygen species
SCNA1: alpha subunit of the voltage-gated sodium ion channel
SLC2A1: solute carrier family 2 member 1 (GLUT1)
SOD1: superoxide dismutase 1
TDP-43: TAR DNA-binding protein 43
TEER: transendothelial electrical resistance
VECAD: VE-cadherin
ZO1: zonula occludens 1
