# Supplementary material for: The role of mutations associated with familial neurodegenerative disorders on blood–brain barrier function in an iPSC model
Source: Fluids Barriers CNS. 2019 Jul 15;16:20. doi: 10.1186/s12987-019-0139-4 (PMC6628493; doi:10.1186/s12987-019-0139-4)
Supplement: Supplementary file 1 — Additional file 1: Figure S1. Phase images of differentiation of iPSCs. Figure S2. Western blots. Figure S3. Expression of individual proteins and genes across all disease lines. Figure S4. Protein and gene expression by disease. Figure S5. Gene expression normalized to WT2 and iPS12 lines. Figure S6. Additional stains: ZO-1. Figure S7. Additional stains: occludin. Figure S8. Additional stains: claudin-5. Figure S9. Additional stains: Glut-1. Figure S10. Cell area and fraction of frayed junctions. Figure S11. Rhodamine 123 permeability. Figure S12. Transport dysfunction by disease. [file 12987_2019_139_MOESM1_ESM.pdf]

The role of mutations associated with familial neurodegenerative disorders on blood-brain barrier function in an iPSC model

Moriah E. Katt,[1,2] Lakyn N. Mayo,[1,2] Shannon E. Ellis,[3] Vasiliki Mahairaki,[4,5] Jeffrey D. Rothstein,[6] Linzhao Cheng,[5] Peter C. Searson[1,2]*

Figure S1: Phase images of differentiation of iPSCs
Figure S2: Western blots
Figure S3: Expression of individual proteins and genes across all disease lines
Figure S4: Protein and gene expression by disease
Figure S5: Gene expression normalized to WT2 and iPS12 lines
Figure S6: Additional stains: ZO-1
Figure S7: Additional stains: occludin
Figure S8: Additional stains: claudin-5
Figure S9: Additional stains: Glut-1
Figure S10: Cell area and fraction of frayed junctions.
Figure S11: Rhodamine 123 permeability
Figure S12: Transport dysfunction by disease

Figure S1: Phase contrast images of differentiation of iPSCs

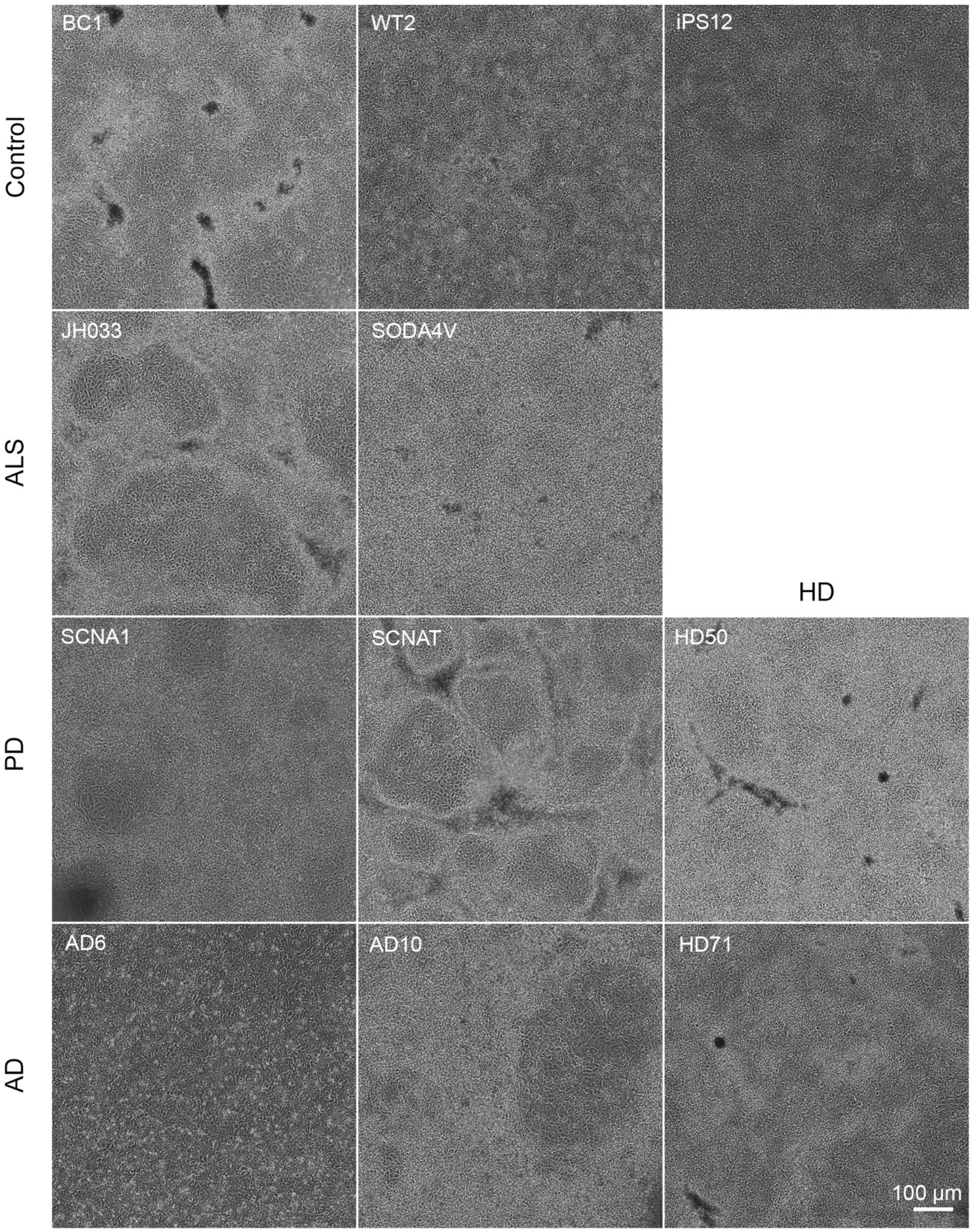

Phase images on day 7 of the differentiation, after the first 24 hours in RA-containing medium. All cell lines show similar characteristics: flat endothelial-like regions surrounded by regions of neural tracts. Neural tracts show variation in thickness, optical density, and height although these variations are within the range of normal differences that are visible within differentiations. For example, the AD6 line shows relatively flat neural tracts which makes them difficult to distinguish under phase microscopy. In contrast, the AD10 line shows large thick neural tracts. When the differentiation is transitioned to RA-containing medium the neural tracts rapidly recede and decrease in size. The shrinking of neural tracts can occur at different speeds or in different ways. In the BC1 cell line, the neural tracts, while relatively transparent, are no longer continuous. The SCNAT line, shows a cell appearance very similar to the BC-1 line, however, all of these differentiations produced high quality cells with high TEER. In cases where the neural tracts are not clearly visible, or are non-ideal in morphology, cell morphology can provide the necessary cues that the correct cell subtypes are differentiating in the correct density, since the cells that make up the neural tracts are more elongated than the cobblestone-like endothelial cells. The brightness and contrast have been adjusted uniformly across all images to improve visualization.

Figure S2: Western blots.

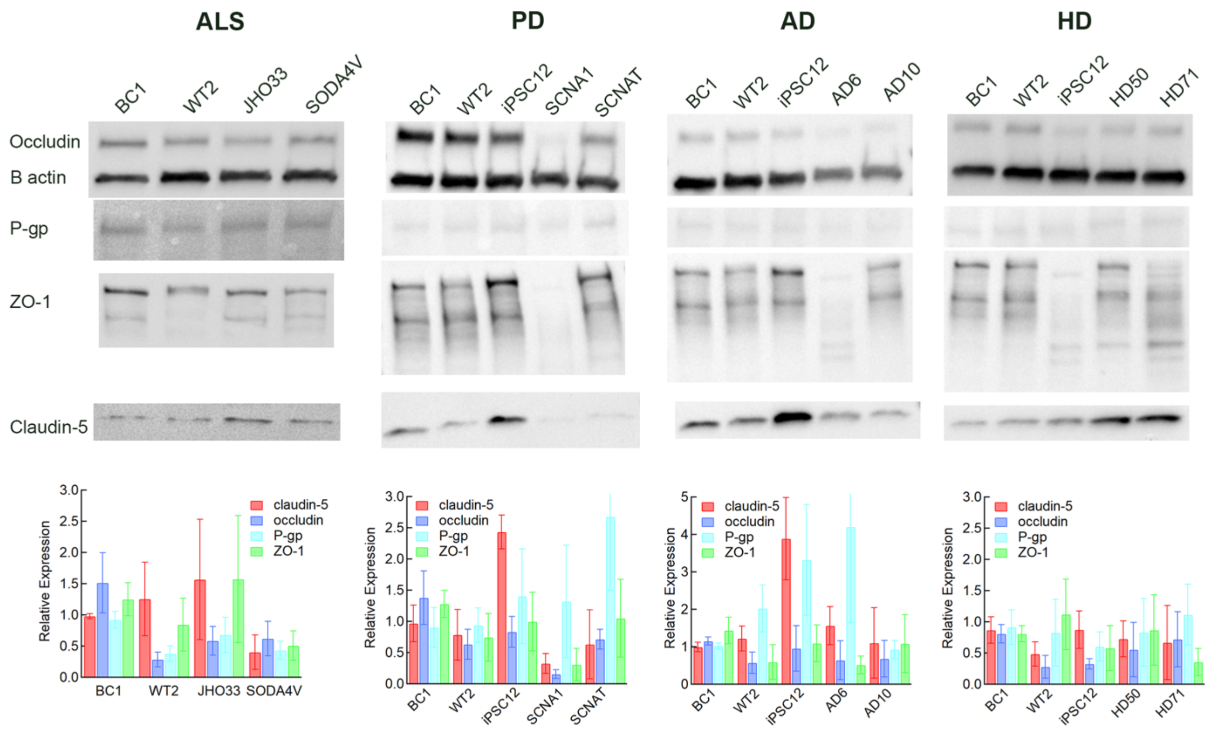

Bands from selected Western blots from all NDD dhBMECs lines. From left to right: ALS, PD, AD, and HD in addition to healthy controls (BC1, WT2, or iPS12). From top to bottom: occludin, beta actin, P-gp, ZO-1, and claudin 5. The bar graphs show the relative expression levels (with respect to β actin) from the Western blots for the cell lines associated with the four diseases. The results included in the main manuscript represent the averages from these four graphs.

Figure S3: Expression of individual proteins and genes across all disease lines.

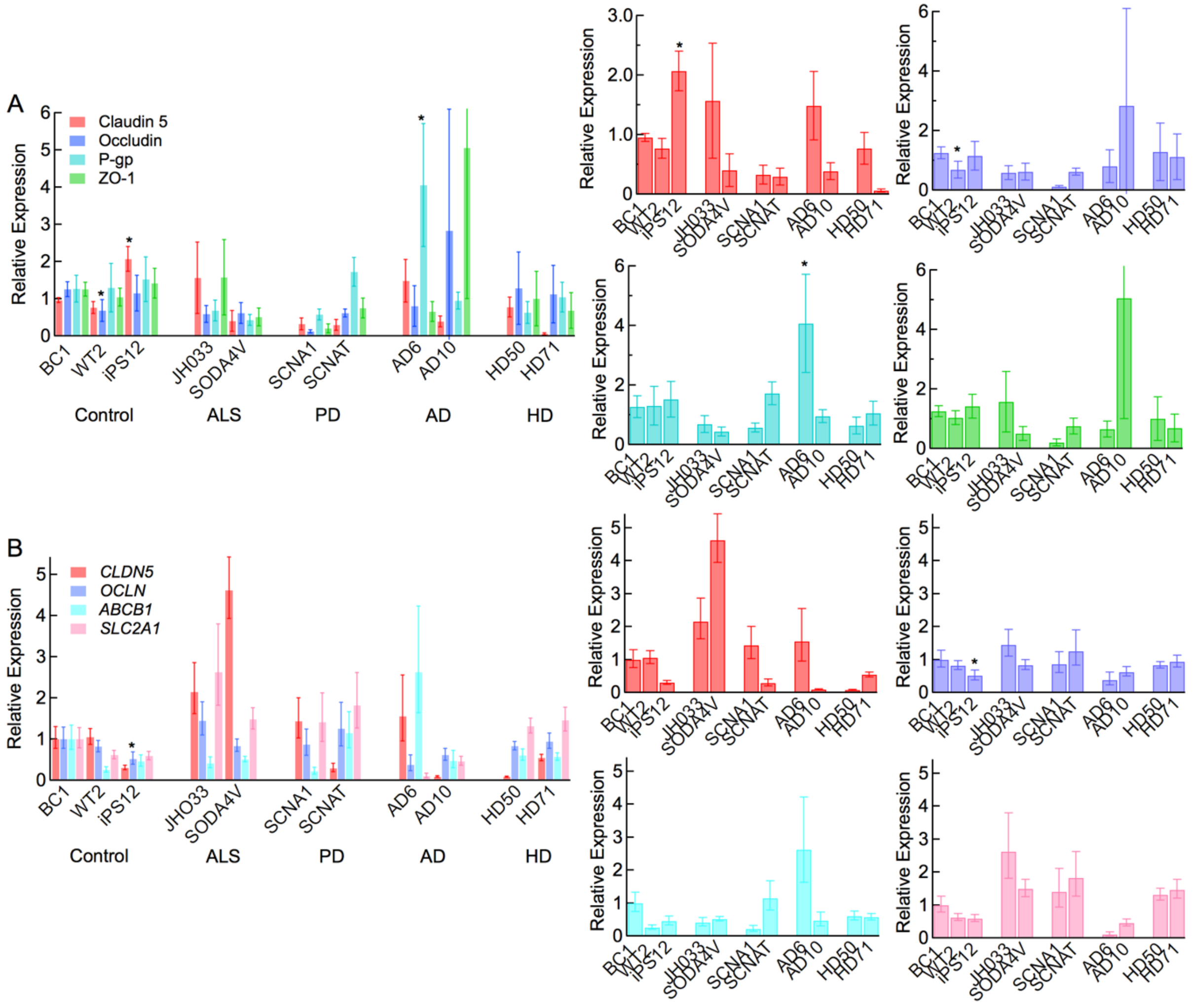

(A) Protein and (B) gene expression graphs from **Figure 1** for individual BBB markers. Data were obtained from 2 technical replicates for each of three differentiations (N = 3). Error bars represent mean ± SE. Statistical analysis was performed using nested ANOVA comparing to the three healthy controls. * $p < 0.05$.

Protein expression: Data were obtained from 2 technical replicates for each of three independent differentiations (N = 3). Results were normalized by first correcting for protein concentration based on β-actin loading control, and then normalized to the first technical replicate of BC1 run on each gel.

Gene expression: Expression levels are normalized to the mean expression level for that gene in the BC1 dhBMEC line after normalization to the housekeeping genes *BACT* and *GPADH*. Data were obtained from 3 technical replicates for each of three differentiations (N = 3).

Figure S4: Protein and gene expression by disease.

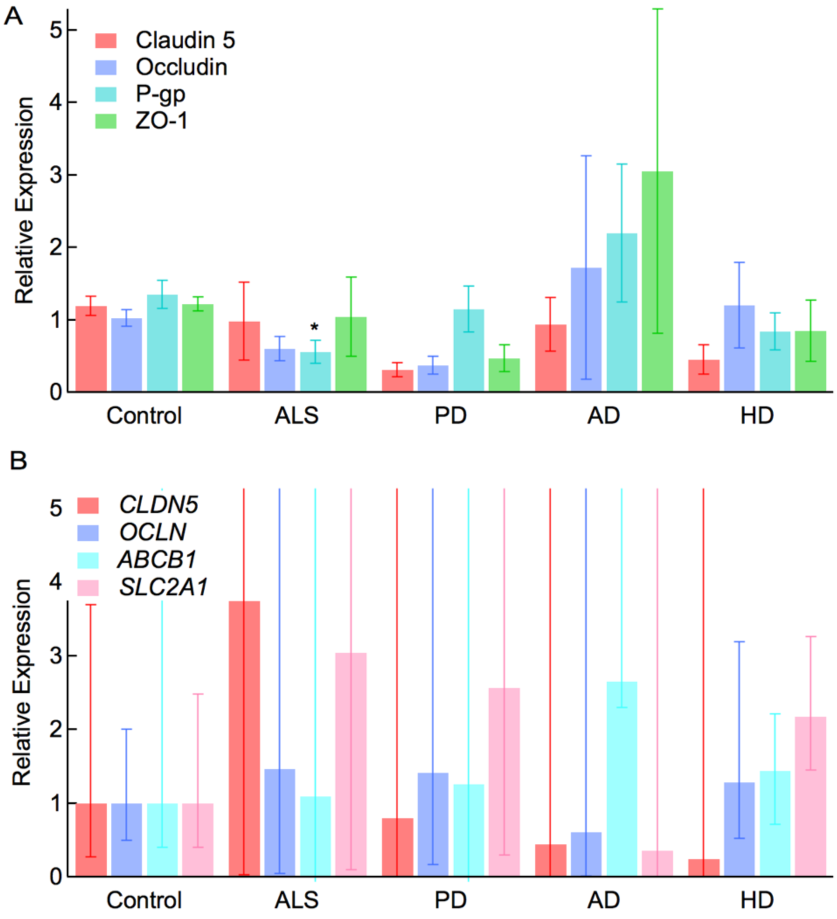

Relative (A) protein and (B) gene expression for each disease normalized to the average values for the three healthy controls (BC1, WT2, and iPS12). The values for each disease represent the average of the two lines associated with that disease. Data were obtained from 2 technical replicates for each of three differentiations (N = 3). Error bars represent mean ± SE. Statistical analysis was performed using nested ANOVA comparing to the three healthy controls. * $p < 0.05$.

Figure S5: Protein and gene expression normalized to WT2 and iPS12 lines

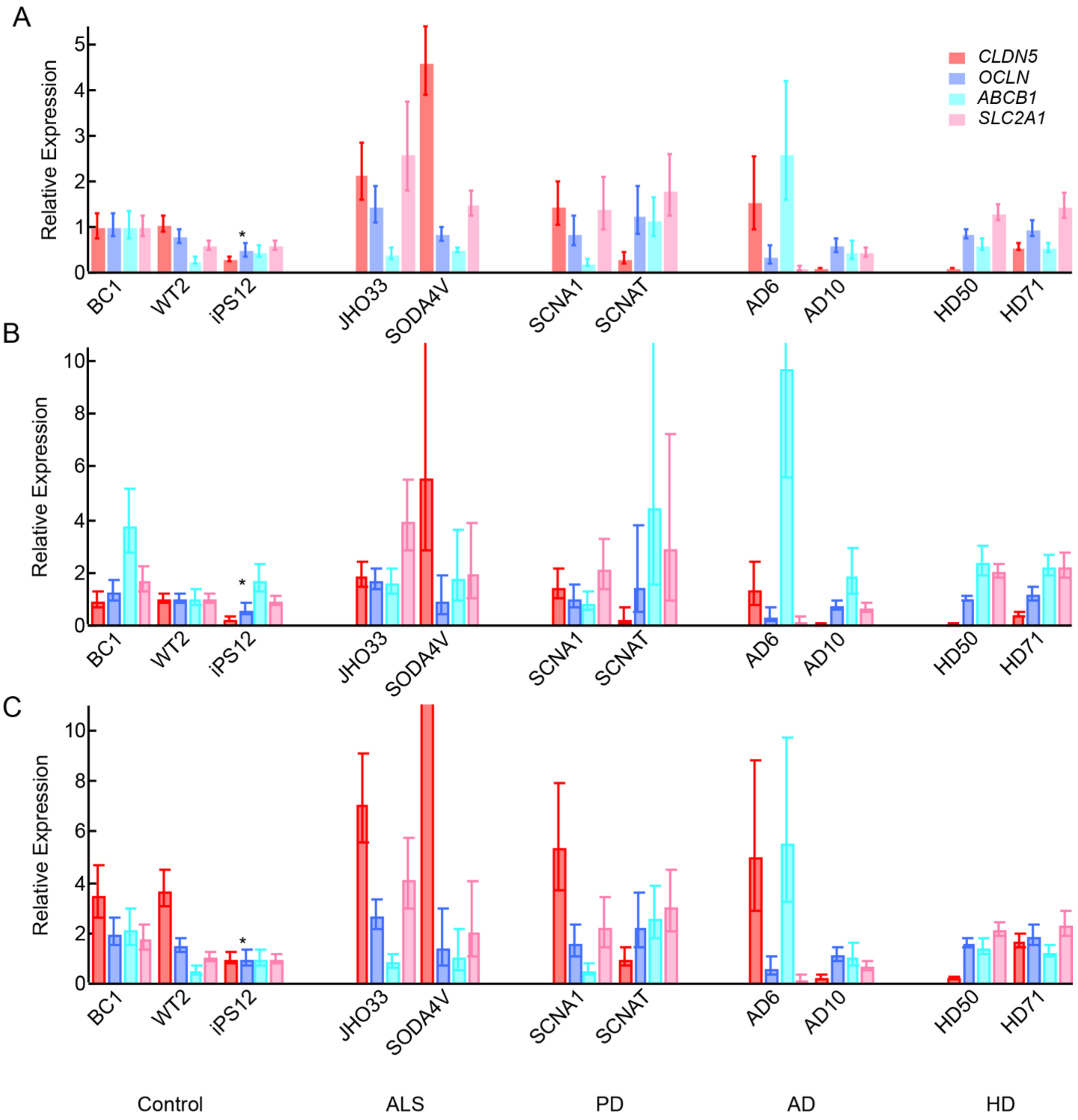

PCR normalized to the three different healthy control lines BC1 (A), WT2 (B), and iPS12 (C). Statistical analysis was performed using nested ANOVA comparing to the three healthy controls. * $P < 0.05$

Figure S6: Additional stains: ZO-1

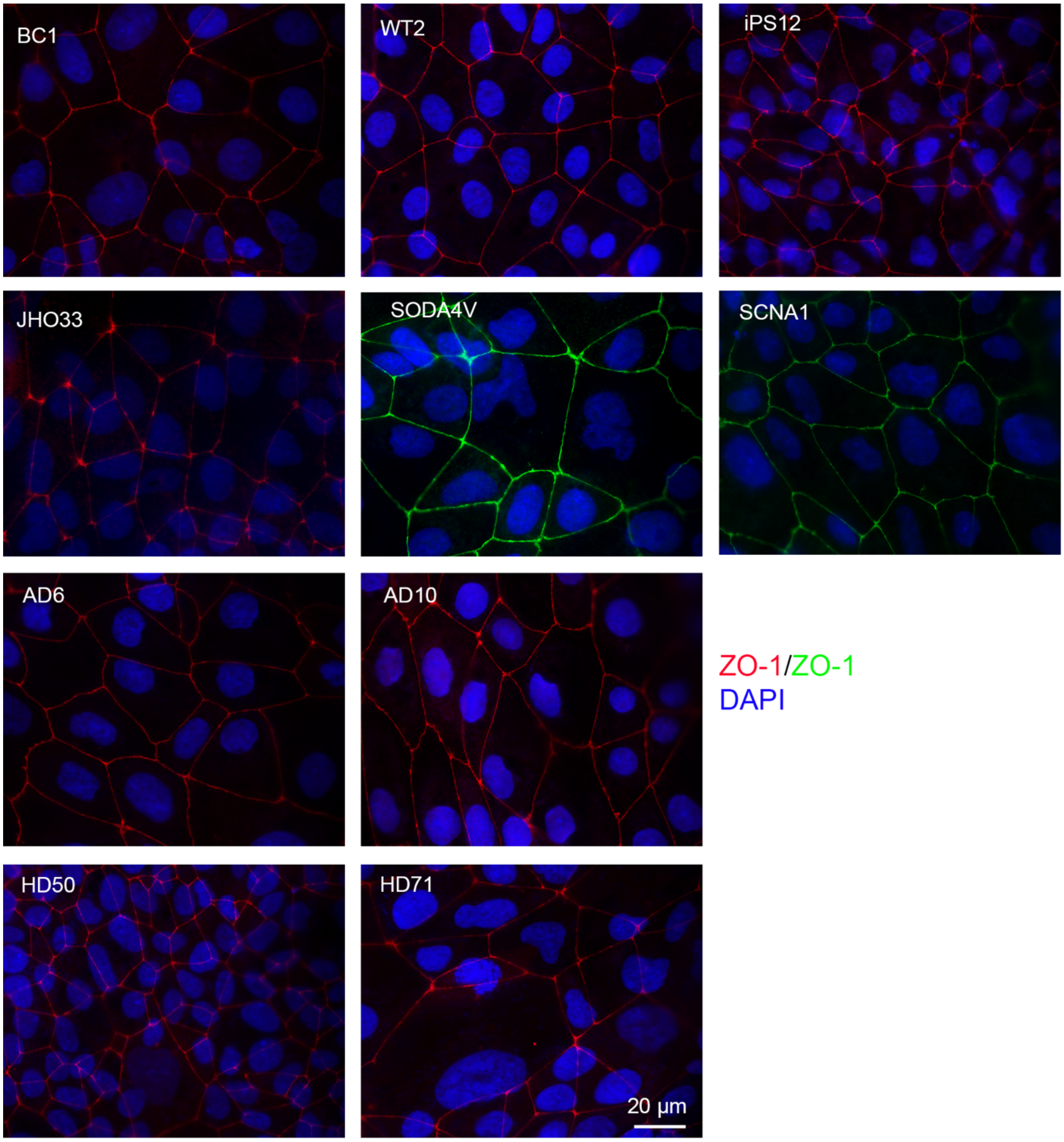

Immunofluorescence images of ZO-1 in NDD cell lines. All cell lines show continuous cell-cell junctions.

Figure S7: Additional stains: occludin

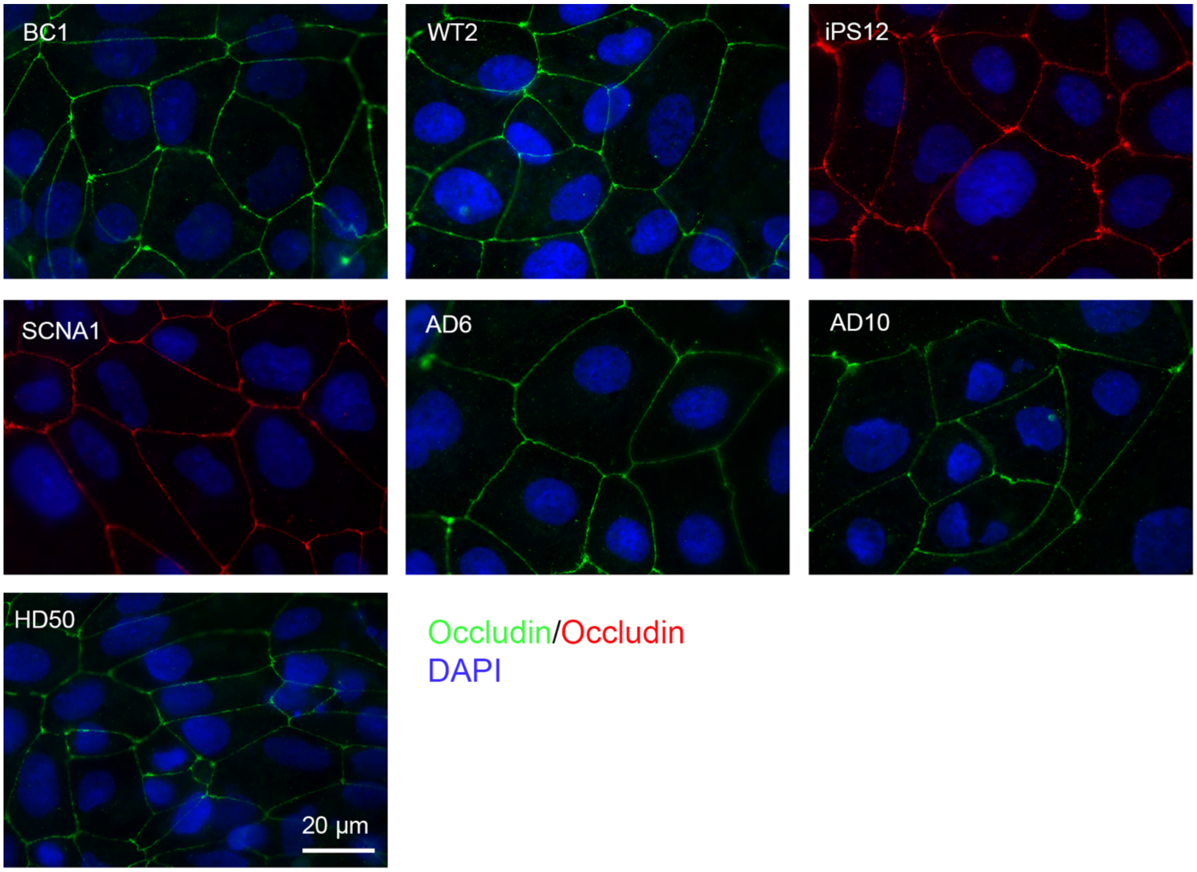

Immunofluorescence images of occludin in selected NDD cell lines. All cell lines show continuous junctions.

Figure S8: Additional stains: claudin-5

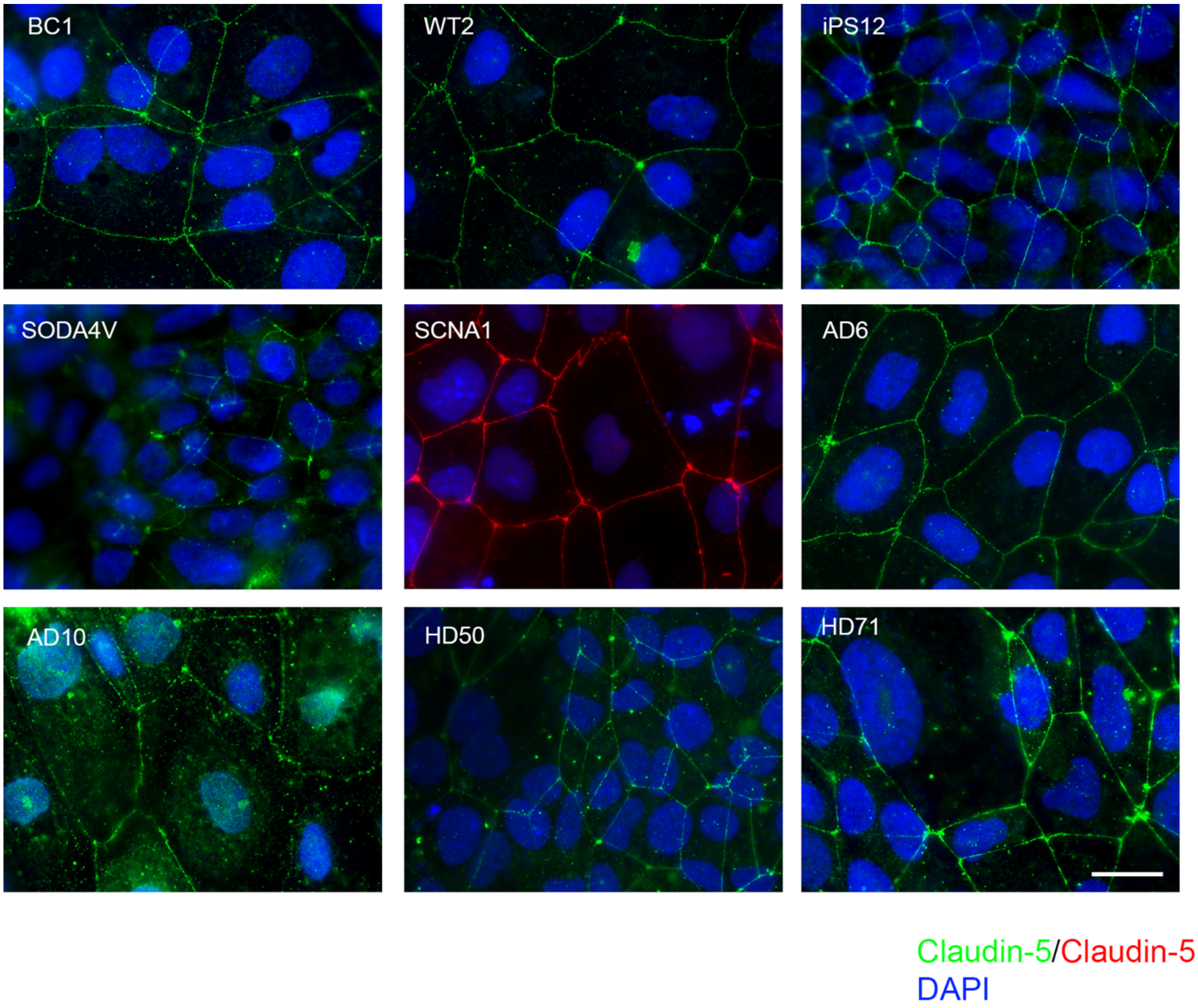

Immunofluorescence images of claudin-5 in NDD cell lines. All cell lines show continuous junctions, with varying degrees of intracellular staining.

Figure S9: Additional stains: Glut-1

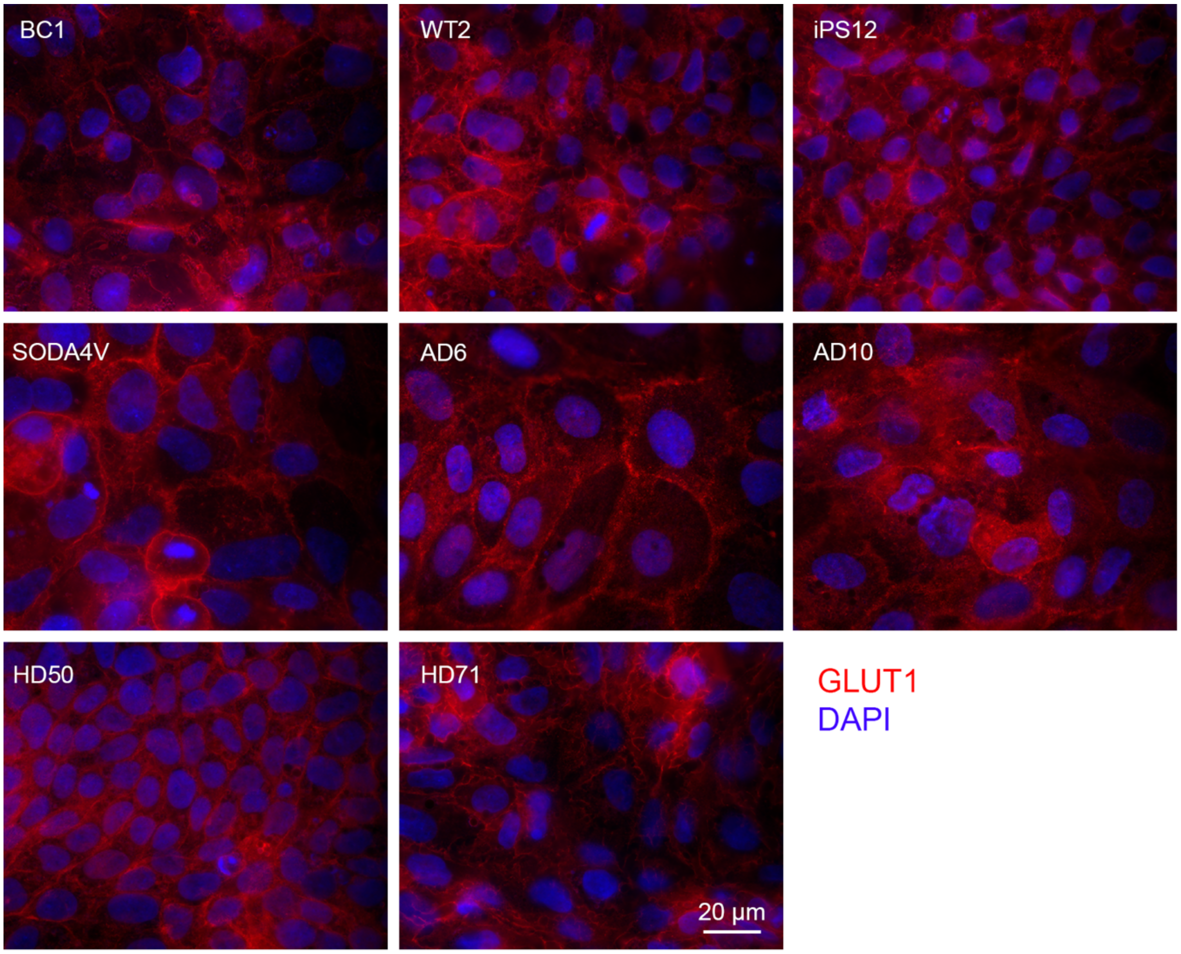

Immunofluorescence images of GLUT1 in the NDD cell lines. All cell lines show global staining, with little variation.

Figure S10: Cell area and fraction of frayed junctions.

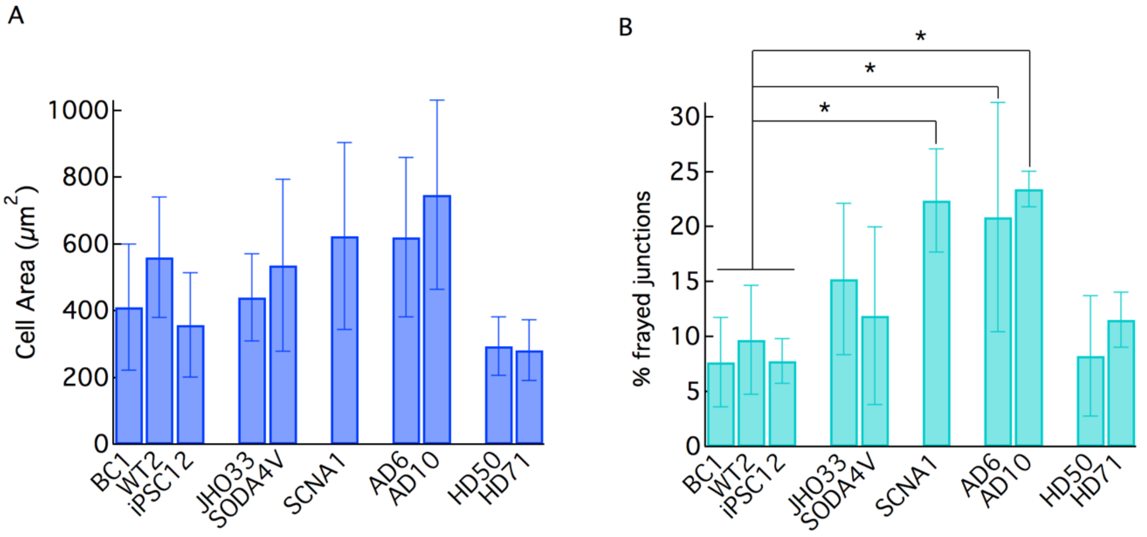

(A) Cell areas were measured for NDD lines from immunofluorescence images of ZO-1 stains with at least 60 cells from each cell line. None of the NDD lines had significantly different cell areas from the healthy controls, with the cells showing some variability across separate differentiations and regions on the same plate. (B) Percent of frayed junctions were measured from immunofluorescence images of claudin-5 with at least 50 cells from each line. Images were analyzed in ImageJ, the total number of junctional segments that showed significant deviation from the straight junctional stain anticipated in a typical tight junction relative to the total number of junctional segments in the image. Increases in % frayed junctions do not overlap with decreases in TEER or paracellular transport. Statistical analysis was performed using nested ANOVA comparing to the three healthy controls. * $p < 0.05$.

Figure S11: Rhodamine 123 permeability.

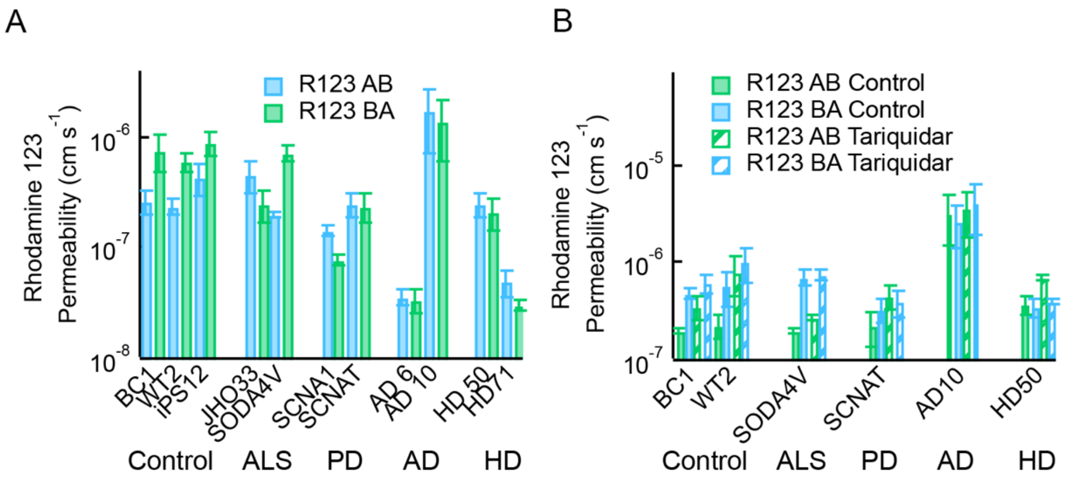

Apical to basolateral (AB) and basolateral to apical (BA) permeability of rhodamine-123: (A) without and (B) with the addition of the P-gp inhibitor Tariquidar.

Figure S12: Transport dysfunction by disease.

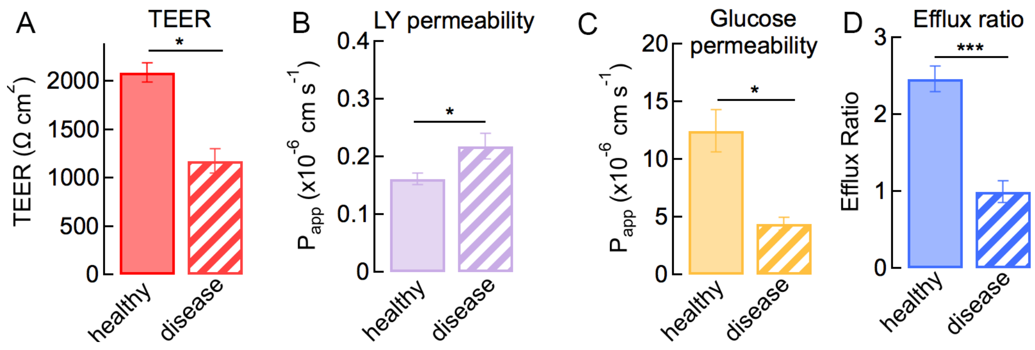

Comparison of barrier function for grouped disease lines and grouped controls. TEER measurements for healthy lines (N = 13 independent differentiations), and disease lines (N = 30 independent differentiations). Lucifer yellow permeability for healthy lines (N = 9, 3 independent differentiations), and disease lines (N = 24 independent differentiations). Glucose permeability for healthy lines (N = 10 independent differentiations), and disease lines (N = 26 independent differentiations). Efflux ratio for rhodamine 123 permeability for healthy lines (N = 14 independent differentiations), and disease lines (N = 33 independent differentiations). Statistical analysis was performed using ANOVA.* $p < 0.05$, *** $p < 0.001$.

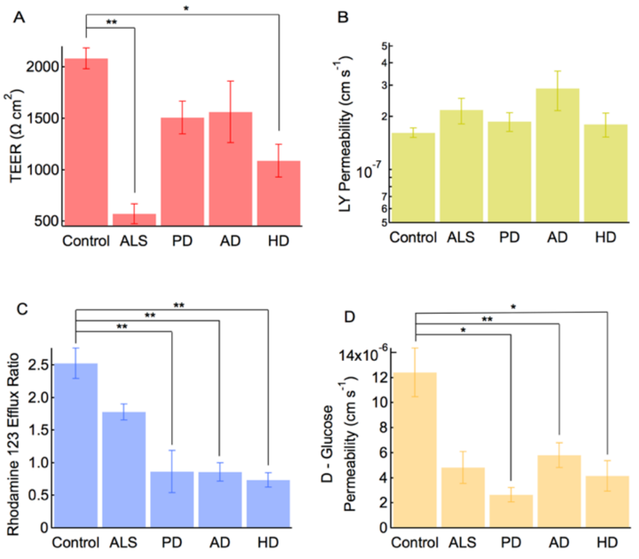

Comparison of barrier function for paired disease lines and grouped controls. Functional transport measurements averaged across the three healthy controls (n = 9) and paired disease lines (n = 6). (A) TEER, (B) Lucifer yellow permeability, (C) R123 efflux ratio, and (D) glucose permeability. Glucose permeability gains statistical significance when grouped into diseases due to the larger replicate number. Statistical analysis was performed using nested ANOVA comparing to the grouped healthy controls.
